# Low-Density Cardoon (*Cynara cardunculus* L.) Particleboards Bound with Potato Starch-Based Adhesive

**DOI:** 10.3390/polym12081799

**Published:** 2020-08-11

**Authors:** Sandra Monteiro, Lina Nunes, Jorge Martins, Fernão D. Magalhães, Luísa Carvalho

**Affiliations:** 1 LEPABE-Faculdade de Engenharia, Universidade do Porto, Rua Dr. Roberto Frias, 4200-465 Porto, Portugal; sandram@fe.up.pt (S.M.); fdmagalh@fe.up.pt (F.D.M.); 2 LNEC (Laboratório Nacional de Engenharia Civil), Structures Department, Av. do Brasil, 101, 1700-066 Lisbon, Portugal; linanunes@lnec.pt; 3 Centre for Ecology, Evolution and Environmental Changes, Azorean Biodiversity Group and University of the Azores, 9700-042 Angra do Heroísmo, Portugal; 4 DEMAD–Department of Wood Engineering, Polytechnic Institute of Viseu, Campus Politécnico de Repeses, 3504-510 Viseu, Portugal; jmmartins@estgv.ipv.pt

**Keywords:** Low-density particleboards, starch-based adhesive, cardoon, physico-mechanical properties

## Abstract

In the present work, and for the first time, totally biosourced low-density particleboards were produced using cardoon particles (a no added value by-product from the Portuguese cheese making industry), bound with a potato starch adhesive. Different starch/cardoon ratios (0.6, 0.8, 1 and 1.2) were tested and the effect of different bio-based additives (chitosan, wood fiber and glycerol) on the performance of the adhesive system was evaluated. The best result was obtained for a formulation with a starch/cardoon mass ratio of 0.8, a chitosan/starch mass ratio of 0.05 and a water/starch mass ratio of 1.75. The particleboards produced had a density of 323 kg·m^−3^, internal bond strength of 0.35 N·mm^−2^ and thickness swelling of 15.2%. The values of density and internal bond strength meet the standard requirements of general-purpose lightweight boards for use in dry conditions according to CEN/TS 16368 specification. Moreover, the susceptibility of the formulations with best results was established against subterranean termites and one decay fungi.

## 1. Introduction

Particleboards are wood-based panels produced from wood particles and/or other lignocellulosic material, bonded with an adhesive system under pressure and heat. Particleboards with a density below 600 kg·m^−3^ are called lightweight particleboards and are usually used in furniture and flooring systems [1].

In general, the adhesives used in the production of particleboards are based in formaldehyde in combination with urea and/or melamine or phenol, due to their good adhesive performance, high reactivity and lower price [2,3,4]. However, the adhesive industry has been searching for alternatives that are more sustainable, eco-friendly and less dependent of fossil resources, while guaranteeing good bonding performance and economic viability [5].

Starch has been one of the most studied natural products due to its low cost, biodegradability, renewability and good binding properties, in particular for cellulosic substrates [6,7,8,9]. The Europe Union produces 9.3 million tons of starch, where 58% in food, 2% in feed and 40% in non-food applications (the majority for paper-related products [10]. Potato starch is presented as large granules (<110 mm) with an ellipsoidal shape. It has higher swelling capability and solubility than other starches and low gelatinization temperature (60–72 °C) [11,12].

**Figure 1:**
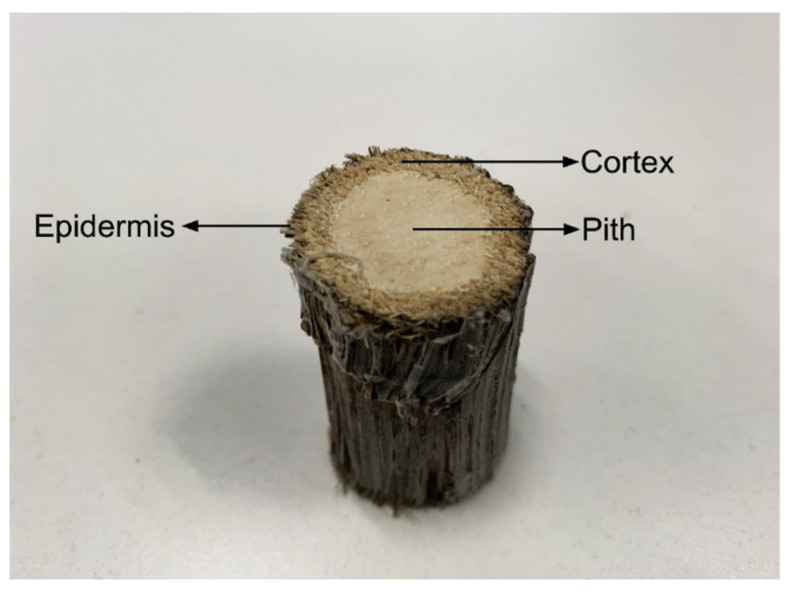
Stalks fraction of cardoon: pith, cortex and epidermis.

Cardoon (*Cynara cardunculus* L.) is native to the Mediterranean basin and belongs to Asteraceae Dumortier family. Its main components, cellulose and lignin, are present in varying amounts, depending on the part of the plant considered [13]. The aqueous extracts from its flowers have been used for centuries as coagulant in the traditional manufacture of ewes’ milk cheese making, due to high content of aspartic proteases and high milk-clotting activity. Cardoon extract confers particular texture and flavor to cheese products. Its use as coagulant in the production of some Portuguese and Spanish traditional ewes’ milk cheeses is required by specific regulations for cheeses that have protected designations of origin [14,15,16]. Even though this is its major current use, cardoon is a crop with a wide range of applications such as human nutrition (due to high amount of nutraceutical and bio-active compounds) [17] and medicine (compounds with anti-tumor, antibacterial and anti-HIV activity can be extracted from the plant) [18]. Cardoon can also be used for the production of lignocellulosic biomass and seed oil, for animal feed and as a raw material in paper pulp and plywood [11,19,20]. In Portugal, cardoon plants are cultivated mostly for the value of the flowers, used in the cheese industry. The stalks and branches are a by-product with no added value. The stalks (Figure 1) have a central pith surrounded by cortex and an irregular thin epidermis [13]. Cardoon stalks have remarkably low-density, which has been determined be several authors. Gominho et al. [21] obtained a density of 208 kg·m^−3^ for stalk particles with 20–30 mm. The authors further determined densities of 218 kg·m^−3^ for depithed stalks (longitudinally section of stalk without pith) and 75 kg·m^−3^ for the pith material. Quilhó et al. [22] determined a density of 138 kg·m^−3^ for whole stalks and 160 kg·m^−3^ for the depithed stalks. Gil et al. [23] reported a density of 114 kg·m^−3^ for milled stalks and branches.

In a previous work, we reported low-density particleboards using pine wood particles bound with a foamable sour cassava starch formulation. The starch foam formed during hot-pressing allowed for intercalation of the wood particles with a low-density but resistant foam, thus providing low-density and good cohesion to the panel. The best results were achieved for particleboards with internal bond strength of 0.67 N·mm^−2^, thickness swelling of 8.7% and a density of 318 kg·m^−3^ [24]. The present work, on the other hand, proposes to take advantage of cardoon particles’ naturally low density to produce lightweight particleboards. Obtaining a starch foam is not therefore paramount, allowing for the use of potato starch, of Portuguese origin, instead of sour cassava starch as the binder.

The goal of the present work is therefore to evaluate the viability of producing low-density particleboards with cardoon particles, instead of wood particles and a potato starch-based adhesive. The effect of adding other components to the adhesive system (chitosan, wood fiber and glycerol) is also studied. This type of formulation has been used successfully in our previous works [24,25] but the effect and relevance of each component was studied. It is intended that the panels have low-density (specified here as ≤ 400 kg·m^−3^) and good mechanical properties (internal bond strength ≥ 0.30 N·mm^−2^, according to the standard requirements for general purpose lightweight boards for use in dry conditions (Type LP2)- CEN/TS 16368) [26]. Furthermore, assuming future indoor applications of these boards (Use Classes 1 and 2, EN 335:2013 [27]) the susceptibility of the formulations with best results was established against the most relevant insect deterioration agent, subterranean termites (UC 1 and 2) and one decay fungi (UC2—occasional increase of moisture).

## 2. Materials and Methods

### 2.1. Materials

Potato starch was supplied by Diverembal Lda. (Alcabideche, Portugal). Glycerol (99.59%) and propionic acid (99%) were supplied by José Manuel Gomes dos Santos Lda. (Odivelas, Portugal). Chitosan (molecular weight around 300 kDa, degree of deacetylation > 85% was purchased from Golden-Shell Pharmaceutical Co. Ltd (Zhejiang, China). Cardoon was provided by Casa da Ínsua (Viseu, Portugal) and chipped and sifted at Polytechnic Institute of Viseu (IPV). The fraction retained corresponded to mesh sizes between 0.25 and 4 mm. The moisture content of the particles was 10%. Maritime pine (*Pinus pinaster* Ait.) fibers were provided by Valbopan-Fibras de Madeira S.A (Nazaré, Portugal).

### 2.2. Preparation of Binder

A cassava starch-based binder formulation described in our previous works with pine wood particleboards [24,25] was used as reference for the optimization of the adhesive system in this work. Variable relative amounts of each formulation component were tested—water, starch, chitosan, wood fiber and glycerol. Initially, potato starch was added to distilled water at room temperature. Chitosan solution, at 5 wt % concentration, was prepared by mixing chitosan and propionic acid solution (6 wt %), during 3 h at 60 °C. This solution was added to the starch and water mixture. Finally, maritime pine (*Pinus pinaster* Ait.) fibers and glycerol were added and stirring was maintained for 5 min.

### 2.3. Wettability of Carddon

The determination of the contact angle on the surface of the different cardoon stalk fractions (pith, cortex and epidermis) was done using a mobile surface analyzer—MSA—Kruss (Hamburg, Germany). The analysis was performed at 20 °C. And the test liquid used was distilled water. The drop volume was 2 μL for the cortex and epidermis. The MSA doses the drop automatically, followed by the direct analysis of the contact angle within milliseconds.

### 2.4. Particleboards Production and Characterization

Cardoon particles were blended manually with the adhesive system. All the particleboards produced were prepared with 200 g dry solids content (starch, chitosan, glycerol, fibers and cardoon). Single layer particleboards were hand formed in square aluminum deformable containers, with dimensions of 220 × 220 × 80 mm^3^. A computer controlled laboratory-scale press, equipped with a linear variable displacement transducer (LVDT), pressure transducer and thermocouples, was used to produce the particleboards.

The adhesive/cardoon mixture was placed on the bottom platen and pressed to 16 mm thickness for 60 seconds. After this, the top platen was raised to 22 mm thickness and the panel was maintained in the press for 240 seconds. The press platen temperature was 190 °C. After pressing, panels were dried at (20 ± 2) °C and relative humidity of (65 ± 5)% till constant mass. During this process boards suffer shrinkage, due to loss of water, attaining thickness between of 16 and 20 mm, depending on the adhesive formulation, which results in different final densities. Seven boards were produced for each adhesive formulation tested.

Density measurements were performed according to EN 323 [28]. The samples were square shaped, with side length of 50 mm. Density was calculated using the mass and volume of specimen after drying.

Moisture content was measured according to EN 322 [29]. It is the ratio between the weight loss of a sample, dried in an oven at (103 ± 2) °C till constant mass and the mass of oven dry-board. The specimen had a square shape with 50 × 50 mm^2^.

Determination of tensile strength perpendicular to the plane of the board, also known as internal bond strength, was performed according to EN 319 [30]. The specimen has a square shape with 50 × 50 mm^2^. The test pieces are glued to the metal loading block using a hot-melt glue (ethylene vinyl acetate). The specimen is subject to a tensile force at constant speed until rupture occurs.

Thickness swelling was determined according to the method described in EN 317 [31]. The increase in thickness of a specimen, with 50 × 50 mm^2^, was evaluated after complete immersion in water for 24 h.

Samples (approx. 30 mm × 10 mm × board thickness) were cut from three of the boards with best results (starch/cardoon mass ratio of 0.8; chitosan/starch mass ratio of 0.05; water/starch mass ratio of 1.75, 2 and 2.25). The susceptibility to subterranean termites (*Reticulitermes grassei* Clément) was tested according to an adaptation of EN 117 [32] with smaller test-specimens and 150 termite workers per flask and the “mini-block method” [33] was used to evaluate the preliminary susceptibility to the brown rot fungus *Gloeophyllum trabeum* (Pers.) Murrill.

Maritime pine (*Pinus pinaster* Ait.) untreated solid wood samples with similar dimensions (30 mm × 10 mm × 10 mm) were included as virulence controls on both tests.

All specimens were conditioned at (20 ± 1) °C and relative humidity of (65 ± 5)% for at least 2 weeks prior to testing. The average initial moisture content measured with an extra set of 4 replicates was 13% for maritime pine and 15% for the cardoon boards.

## 3. Results and Discussion

### 3.1. Cardoon Wettability

Contact angles were measured using the sessile drop method. A droplet of water was placed on the surface of the central pith, cortex and epidermis in order to determine relative wettability. The results are shown in Table 1, showing that the cardoon components are hydrophilic and therefore easily wetted by the aqueous binder solution. The somewhat higher contact angle obtained for the central pith may be due to its foamed structure, containing air voids that hinder wetting.

### 3.2. Effect of Starch/Cardoon Ratio

In the present work, different binder compositions were tested for the production of particleboards. Firstly, the impact of different starch/cardoon ratio on the physico-mechanical properties of the panels was evaluated. The starch/cardoon dry mass ratios tested were 0.6, 0.8, 1 and 1.2. The water/starch mass ratio was 2 and chitosan and wood fiber were not present in the formulation at this point.

**Figure 2:**
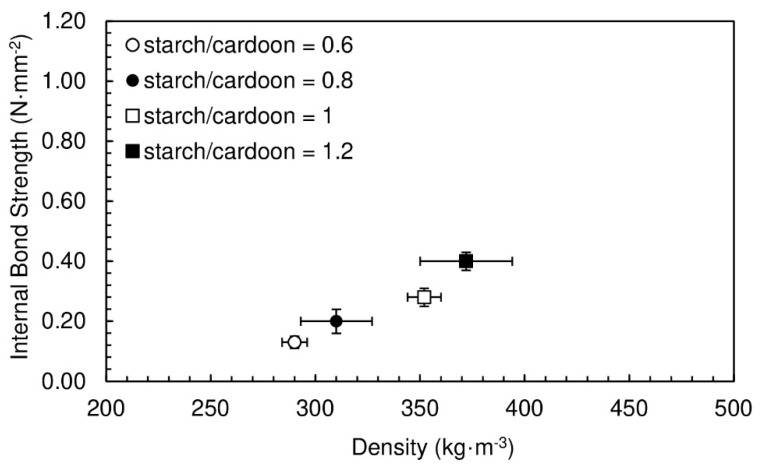
Internal bond strength of particleboards bonded with potato starch as function of density for starch/cardoon ratios of 0.6, 0.8, 1 and 1.2. All formulations had a water/starch ratio of 2.

The particleboards produced had a moisture content between (19.0 ± 0.4)% and (19.1 ± 0.6)%. The internal bond strength and the final densities of the particleboards produced with the different starch/cardoon ratios are shown in Figure 2.

The obtained densities vary between 290 and 372 kg·m^−3^. The lowest densities were achieved for panels produced with the lowest starch/cardoon ratio, 0.6. This is an expected result taking into account that cardoon is less dense than potato starch. A decrease in starch/cardoon ratio leads to a decrease in the particleboard density, since it is increasing the fraction of the component with lower density.

The increase of internal bond strength follows the increase in density, which is expected since higher density implies higher internal cohesion. The internal bond strength values obtained vary between 0.13 and 0.40 N·mm^−2^. The goal of the present work is to produce particleboards with the lowest density possible, maintaining good bond quality (internal bond strength ≥ 0.30 N·mm^−2^. Note that, according to European Technical Specification CEN/TS 16368 [26], the minimum requirement for internal bond of lightweight particleboards type LP2 (the most demanding) with thickness between 13 and 20 mm, is 0.30 N·mm^−2^.

From the results above, only the formulation with starch/cardoon ratio of 1.2 provides intended internal bond strength within this goal.

**Figure 3:**
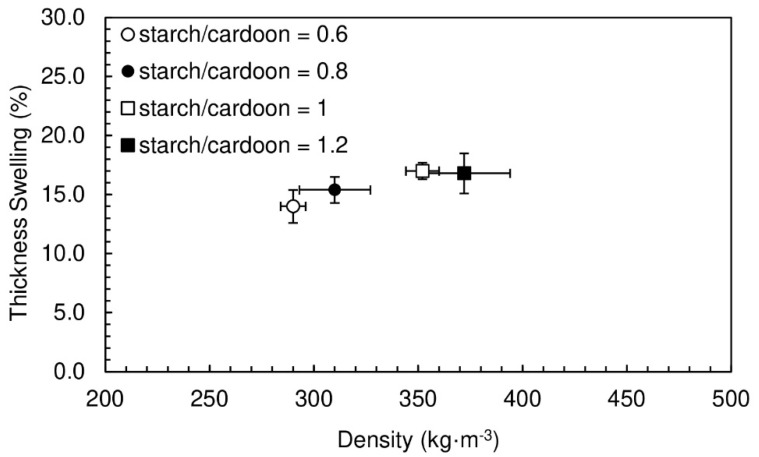
Thickness swelling of particleboards bonded with potato starch for starch/cardoon ratios of 0.6, 0.8, 1 and 1.2. All formulations had a water/starch ratio of 2.

The thickness swelling results are shown in Figure 3, the obtained values vary between 14.0% and 17.0%. As expected, the thickness swelling follows the same trend as density, panels with lower density absorb less water and consequently the swelling tends to be slightly lower. The lower the mass of material per unit volume, the lower will be the amount of water absorbed and thus the lower the swelling. These water swelling results can be considered quite good considering that European standard EN 312 specifies maximum thickness swelling of 14% for use in humid conditions (P3 class), for non-load-bearing boards with thickness between 13 and 20 mm [34]. The particleboards produced in this work are not intended for use humid conditions but still present water swelling values close to the limit allowed for those conditions. Even when comparing with wood-based particleboards produced with synthetic adhesives, the results are remarkable. Pine wood particleboards bounded with urea-formaldehyde resins reported in the literature present thickness swelling values between 27% and 38% [35,36].

### 3.3. Effect of Chitosan Content

Chitosan (cs) is known to have good affinity towards starch, both due to hydrogen bonding and electrostatic interaction between the amine groups of chitosan and the phosphate groups of potato starch [37]. The combination of the two polysaccharides is therefore generally beneficial for the binding performance of starch-based adhesive systems [38]. In addition, chitosan may also establish hydrogen bonding with the lignocellulosic cardoon components [39].

**Figure 4:**
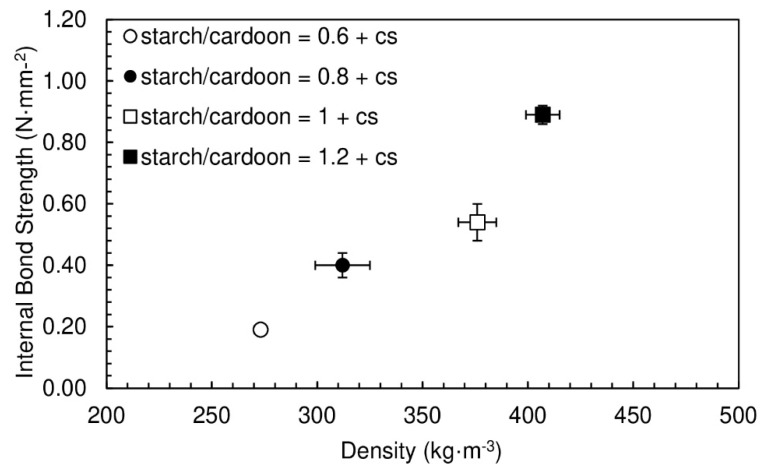
Internal bond strength of particleboards bonded with potato starch as function of density for starch/cardoon ratios of 0.6, 0.8, 1 and 1.2. All formulations had chitosan/starch ratio of 0.05 and water/starch ratio of 2.

Chitosan was added to the previous formulations in order to obtain chitosan/starch ratios of 0.05. The results of internal bond strength and the densities for the particleboards produced are shown in Figure 4. The panels had moisture content between (16.4 ± 0.1)% and (19.2 ± 0.5)%.

The densities vary between 273 and 407 kg·m^−3^, which is similar to the range obtained for particleboards produced without chitosan. On the other hand, a significant improvement is observed in internal bond strength—the values are, in some cases, about twice the ones obtained without chitosan. This confirms the predicted positive effect of the polysaccharide in the particleboard’s cohesion. The internal bond strength is observed to increase with density, as expected.

**Figure 5:**
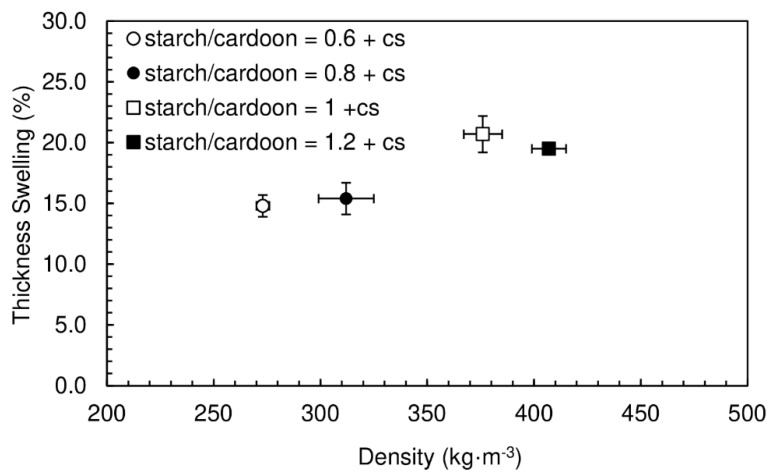
Thickness swelling of particleboards bonded with potato starch for starch/cardoon ratios of 0.6, 0.8, 1 and 1.2. All formulations had chitosan/starch ratio of 0.05 and water/starch ratio of 2.

The thickness swelling results are shown in Figure 5. The thickness swelling values vary between 14.8% and 20.7%, being similar to the ones previously obtained with starch alone.

Starch/cardoon ratios of 0.6 and 0.8 were selected to the ensuing studies, since these yielded the lowest densities, with mechanical resistances around the target threshold of 0.30 N·mm^−2^.

### 3.4. Effect of Fibre Content

Wood fiber has been reported as being an effective reinforcing filler for starch foam [40]. In the current work particleboards were produced with starch/cardoon ratios of 0.6 and 0.8, with and without chitosan addition and fiber/starch ratio of 0.05. A ratio of 0.1 was also tested, however the high amount of fiber increases the viscosity of the adhesive, hindering its dispersion significantly. As a consequence, the panels manufactured did not present enough cohesion to justify being subjected to mechanical testing.

**Figure 6:**
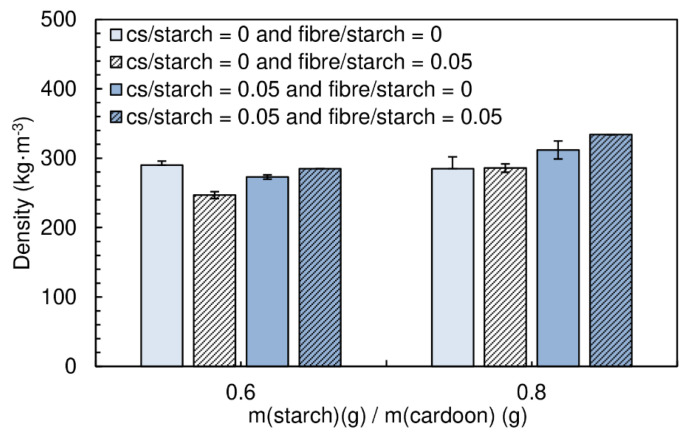
Density of the particleboards produced with fiber and fiber combined with chitosan. All adhesive formulations had a starch/cardoon ratio of 0.6 and 0.8.

The panels produced had a moisture content between (16.9 ± 0.1)% and (20.2 ± 0.2)%. The particleboards with a starch/cardoon ratio of 0.6 exhibit densities between 247 and 290 kg·m^−3^, while those produced with a starch/cardoon ratio of 0.8 had densities between 286 and 334 kg·m^−3^ (Figure 6).

**Figure 7:**
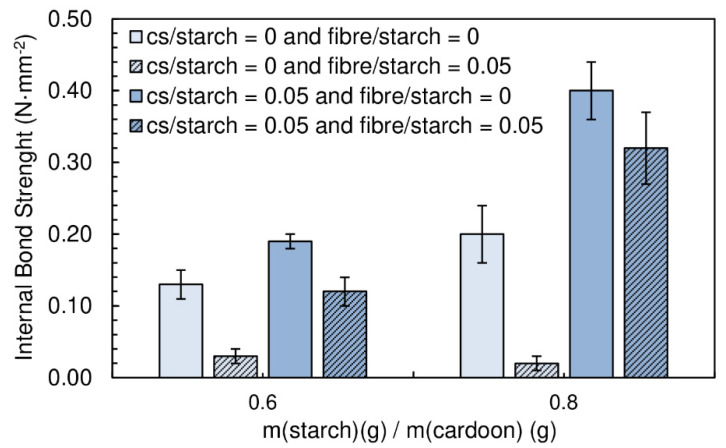
Effect of fiber content and the combined effect of chitosan and fiber on internal bond strength of the particleboards bonded with a potato starch. All adhesive formulations had a starch/cardoon ratio of 0.6 and 0.8.

Figure 7 shows the effect of the content of fiber on the internal bond strength of the panels.

The combination of wood fiber with starch decreases very significantly the internal bond strength of the panels. When chitosan is added, the performance decay is less notorious. Wood fiber is not easily dispersible in the starch suspension, tending to form agglomerates. This has a detrimental effect of mechanical resistance, since these act as defects with low cohesion. Chitosan was seen to facilitate significantly fiber deagglomeration/dispersion. This results from the joint effect of high shear, due to higher viscosity of the medium when chitosan is present and the possible action of chitosan as a surfactant for the fibers dispersion in water. Nevertheless, the combination of chitosan and fibers does not provide better strength than chitosan alone. It must be noted that this binder formulation has very high viscosity and it is difficult to guarantee a homogeneous mixture with the cardoon particles.

**Figure 8:**
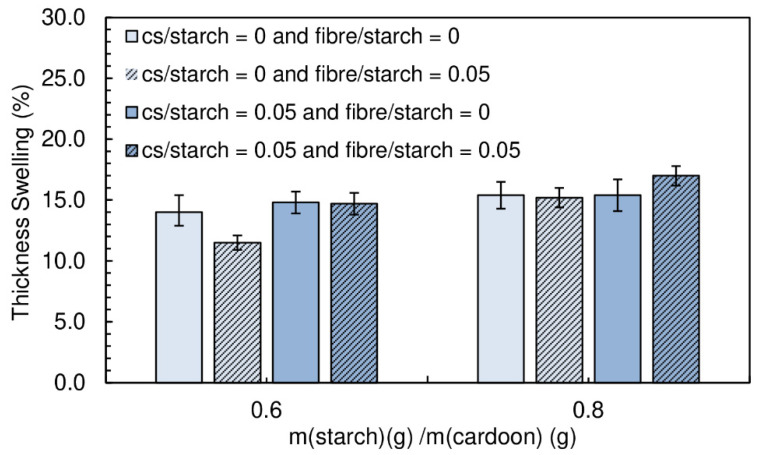
Effect of fiber content and the combined effect of chitosan and fiber on thickness swelling of the particleboards bonded with a potato starch. All adhesive formulations had a starch/cardoon ratio of 0.6 and 0.8.

The thickness swelling results are shown in Figure 8. No significant changes can be observed with the addition of fibers.

### 3.5. Effect of Glycerol Content

Glycerol is an effective plasticizer for starch, as the intermolecular chain interactions (strong hydrogen bonding and chain entanglements) are replaced by stronger interactions with glycerol. This results in higher chain mobility, therefore decreasing stiffness [41,42]. This may benefit internal bond strength—higher flexibility of the binder may allow for slight displacements of the wood particles during the application of stress without causing breakage of interparticular bonds.

**Figure 9:**
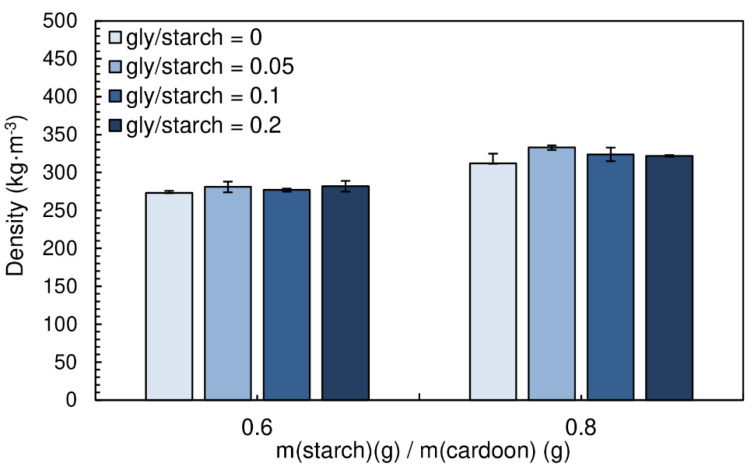
Density of the particleboards produced with different gly/starch ratios. All adhesive formulations had a starch/cardoon ratio of 0.6 and 0.8 and cs/starch ratio of 0.05.

The density of the particleboards produced with different glycerol/starch ratios (0.05, 0.1 and 0.2) are shown in Figure 9. The densities vary between 273 and 282 kg·m^−3^ for starch/cardoon ratio of 0.6, while for panels produced with starch/cardoon ratio of 0.8 the densities vary between 312 and 333 kg·m^−3^.

**Figure 10:**
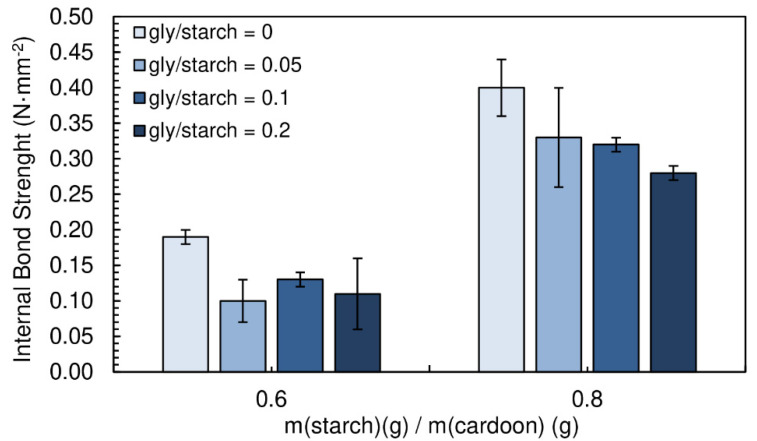
Effect of glycerol content on internal bond strength of the particleboards bonded with a potato starch. All adhesive formulations had a starch/cardoon ratio of 0.6 and 0.8 and cs/starch ratio of 0.05.

Figure 10 presents the influence of the addition of glycerol (gly) on the internal bond strength of the particleboards. A chitosan/starch ratio of 0.05 was maintained for all formulations. The particleboards produced had a moisture content between (16.9 ± 0.1)% and (19.8 ± 0.1)%.

The addition of glycerol had no positive effect on the internal bond strength of the particleboards. In fact, it is possible to observe a decreasing trend on the resistance of the panels. Glycerol molecule, as efficient starch plasticizer, builds between polysaccharide molecules, what results in decreasing brittleness but also tensile strength of the polysaccharide matrix.

**Figure 11:**
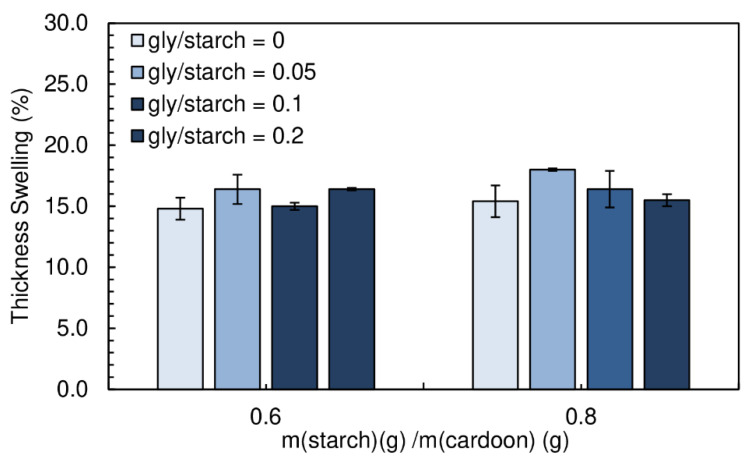
Effect of glycerol content on thickness swelling of the particleboards bonded with a potato starch. All adhesive formulations had a starch/cardoon ratio of 0.6 and 0.8 and cs/starch ratio of 0.05.

The thickness swelling results are shown in Figure 11. Again, no significant influence can be observed with the addition of glycerol.

### 3.6. Effect of Water Content

**Figure 12:**
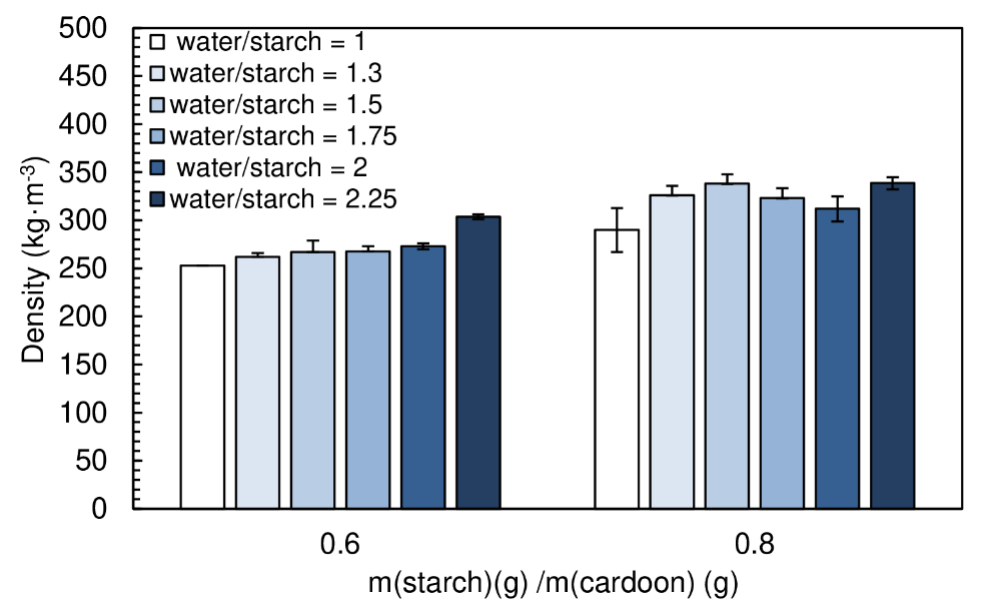
Density of the particleboards produced with different water/starch ratios. All adhesive formulations had a starch/cardoon ratio of 0.6 and 0.8 and cs/starch ratio of 0.05.

Water plays a key role on the binder performance, since it is responsible for starch gelatinization, which is paramount for the adhesion process, so that interaction of starch chains with the particles surface is maximized. On the other hand, too much water lengthens the drying time of the particleboards [25]. Different ratios water/starch were tested (1, 1.3, 1.5, 1.75, 2 and 2.25), the densities of the produced panels are shown Figure 12.

**Figure 13:**
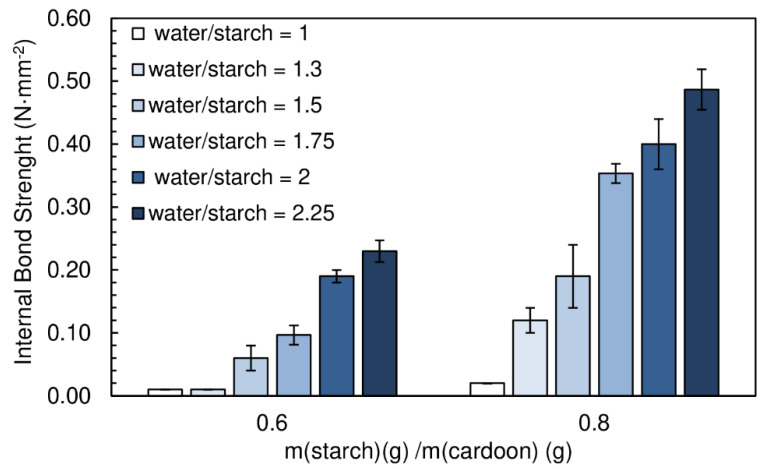
Effect of water content on internal bond strength of the particleboards bonded with a potato starch. All adhesive formulations had a starch/cardoon ratio of 0.6 and 0.8 and cs/starch ratio of 0.05.

Figure 13 presents the effect of water content on the internal bond strength of the panels manufactured. Recall that the previous results were obtained for a water/starch ratio of 2. A chitosan/starch ratio of 0.05 was used in all formulations. The particleboards produced had a final moisture content between (16.9 ± 0.1)% and (19.2 ± 0.5)%.

Bond resistance increases steadily with the amount of water in the adhesive formulation. The best internal bond performance (0.49 N·mm^−2^) was achieved for particleboards with a ratio water/starch of 2.25. This results from the more effective starch gelatinization process under excess water. Complete gelatinization is essential for starch’s crystalline double-helix chains to dissociate, breaking up the granules and therefore exposing the hydroxyl groups for interaction with the substrate.

**Figure 14:**
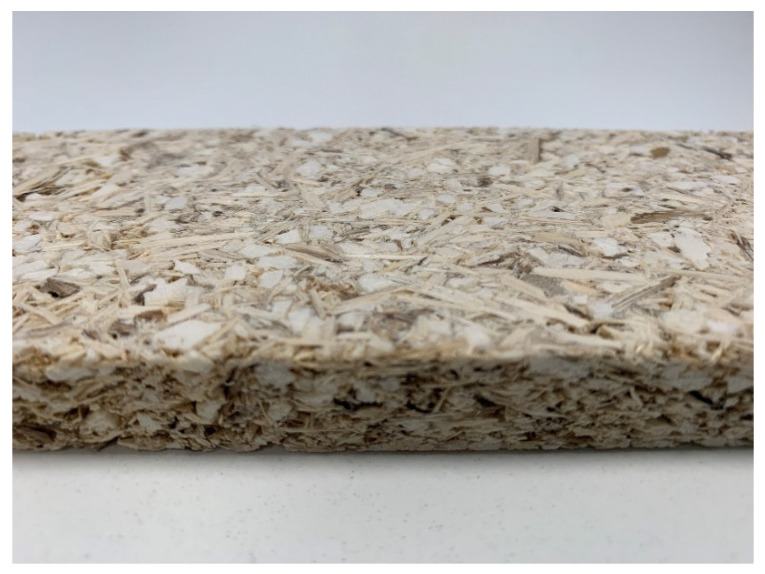
Cardoon particleboard with potato starch binder. Panel thickness is 16 mm.

The particleboards produced with starch/cardoon ratio of 0.8 and water/starch ratio of 2.25 meet the proposed objectives, density ≤ 400 kg·m^−3^) and internal bond strength ≥ 0.30 N·mm^−2^. However, in order to minimize the drying time, the particleboards produced with a water/starch ratio of 1.75 satisfy the goal of this work. An example of a panel produced in these conditions is shown in Figure 14.

**Figure 15:**
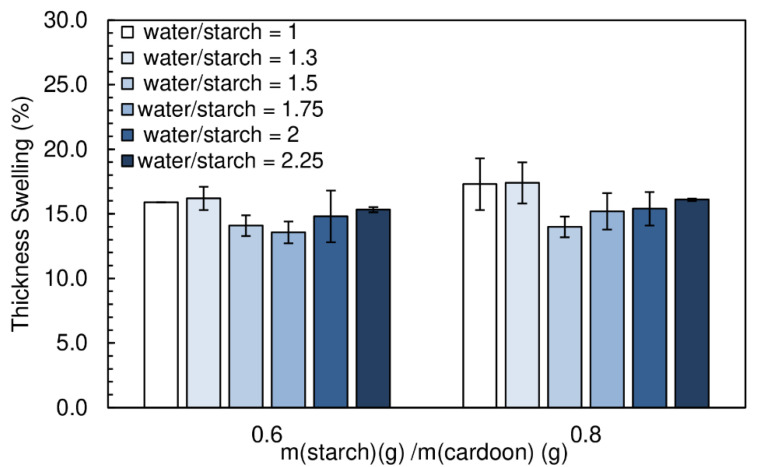
Effect of water content on thickness swelling of the particleboards bonded with a potato starch. All adhesive formulations had a starch/cardoon ratio of 0.6 and 0.8 and cs/starch ratio of 0.05.

Thickness swelling results are shown in Figure 15. The amount of water did not have a significant effect on the thickness swelling of the particleboards. The values obtained ranged between (14.0 ± 0.8)% and (17.4 ± 1.6)%.

The work’s goals were attained with the adhesive formulation contained starch/cardoon ratio of 0.8, a cs/starch ratio of 0.05 and water/starch ratio of 1.75. The particleboards produced with this adhesive formulation have a density of 323 kg·m^−3^ with internal bond strength of 0.35 N·mm^−2^ and thickness swelling of 15.2%.

### 3.7. Susceptibility to Biological Deterioration

#### 3.7.1. Subterranean Termites

As can be seen in Table 2, all tested cardoon particleboard compositions showed high susceptibility to subterranean termites in no-choice test. The high attack rating and survival on the control maritime pine samples, which was expected, validates the test. A significantly higher final moisture content was noted for the cardoon boards when compared to the pine controls. Considering the observed variability, the results indicate that the panels based on cardoon and starch show a level of attack by termites similar to maritime pine wood.

#### 3.7.2. Wood-Decay Fungi

After the six weeks exposure period to *G*. trabeum, the test-specimens were carefully removed from the test flasks and the external visible fungal hyphae cleaned as much as possible. The specimens were then weighed and final dry mass determined by the oven method at 103 °C. The final moisture content and mass loss was calculated and is presented in Table 3. Again, in this test a significantly higher final moisture content was noted for the boards when compared to the pine controls.

In this case, the cardoon/starch panels show higher susceptibility towards fungi attack, yielding about twice the mass loss of maritime pine wood. This is probably a consequence of fungi attack on starch, the starch may serve as nutrition supplement for the fungi.

## 4. Conclusions

This work proposes the use of cardoon particles bound with potato starch adhesive for production of lightweight particleboards appropriate for interior furniture. The aim was to produce totally biosourced panels with density below 400 kg·m^−3^ and internal bond strength of at least 0.30 N·mm^−2^.

The particleboards produced with starch/cardoon ratios ranging from 0.6 to 1.2 had densities between 290 and 372 kg·m^−3^. Density increases with the relative amount of potato starch. The internal bond strength follows the increase of density. The values obtained varied between 0.13 and 0.40 N·mm^−2^.

The addition of chitosan to the starch adhesive leads to a notorious improvement in internal bond strength of the panels for all starch/cardoon ratios studied (0.19 to 0.89 N·mm^−2^), with practically no change in density. The enhancement in mechanical properties may result from electrostatic interaction between amine groups of chitosan and phosphate groups of starch, as well as from hydrogen bonding between chitosan and cellulose.

The introduction of wood fiber in the formulation was detrimental to the mechanical properties of the particleboards due to poor dispersibility of fibers. The presence of chitosan was seen to improve the dispersion and consequently the mechanical performance of the panels. However, better results were obtained with chitosan alone, so it was concluded that the fibers do not provide a reinforcement effect in this system.

Glycerol, which is a well-known plasticizer for starch, was tested, since a reduction in the stiffness of the binder could be beneficial for the particleboards’ mechanical resistance. However, no improvement was observed.

The amount of water proved to be an important component in the adhesive system. The internal bond strength of the cardoon panels improved with the increase of the water to starch ratio, probably due to more effective starch gelatinization, leading to better binder cohesion.

The starch-based panels showed the same behavior towards termite attack as maritime pine wood. On the other hand, exposure to fungi caused about two times more mass loss on the composite panels than on the reference maritime pine, certainly due to starch’s susceptibility towards microbial attack.

The best adhesive formulation consisted of a starch/cardoon ratio of 0.8, chitosan/starch ratio of 0.05 and water/starch ratio of 1.75. The particleboards produced with this adhesive formulation presented density of 323 kg·m^−3^, with an internal bond strength of 0.35 N·mm^−2^ and thickness swelling of 15.2%. The combination of cardoon particles with starch-based binder is therefore a promising biosourced solution for low-density particleboards appropriate for interior furniture where the problems related to possible biological deterioration are minimized.

## Figures and Tables

**Table 1:** Contact angle of different parts of the cardoon stalks.

Part of Cardoon Plant	Contact Angle (°)
Central pith	67 ± 9
Cortex and epidermis	46 ± 9

**Table 2:** Average results of survival, mass loss (% of initial dry mass), final moisture content and grade of attack (from 0-no attack, to 4-heavy attack) of particleboards exposed to *R. grassei* (*n* = 3). All adhesive formulations had a starch/cardoon ratio of 0.8 and cs/starch ratio of 0.05.

Board/Wood	Water/Starch Ratio	Final Moisture Content (%)	Survival (%)	Average Mass Loss (%)	Average Grade of Attack
cardoon/starch	1.75	66.54 ± 17.35	70.89 ± 15.78	16.46 ± 10.43	4
cardoon/starch	2	68.08 ± 10.70	83.78 ± 2.41	14.15 ± 2.71	4
cardoon/starch	2.25	79.40 ± 16.62	53.11 ± 29.17	15.29 ± 9.71	4
Maritime pine control	-	38.60 ± 19.06	85.11 ± 5.18	12.01 ± 1.87	4

**Table 3:** Average results of mass loss (% of initial dry mass) and final moisture content of the particleboards bonded with a potato starch and their paired maritime pine controls exposed to *G. trabeum* (*n* = 10). All adhesive formulations had a starch/cardoon ratio of 0.8 and cs/starch ratio of 0.05.

Board/Wood	Water/Starch Ratio	Final Moisture Content (%)	Mass Loss (%)
cardoon/starch	1.75	185.89 ± 62.15	30.76 ± 6.56
Maritime pine control	-	44.56 ± 5.64	12.70 ± 6.30
cardoon/starch	2	169.70 ± 34.95	25.56 ± 2.96
Maritime pine control	-	44.64 ± 5.29	16.98 ± 7.23
cardoon/starch	2.25	173.63 ± 32.11	29.39 ± 4.94
Maritime pine control	-	47.71 ± 7.77	15.14 ± 8.29
